# Development, acceptability and uptake of an on-line communication skills education program targeting challenging conversations for oncology health professionals related to identifying and responding to anxiety and depression

**DOI:** 10.1186/s12913-022-07521-5

**Published:** 2022-01-31

**Authors:** Joanne Shaw, Karen Allison, Jessica Cuddy, Toni Lindsay, Peter Grimison, Heather Shepherd, Phyllis Butow, Tim Shaw, Kate Baychek, The ADAPT Program Group, Karen Allison, Gavin Andrews, Kate Baychek, Philip Beale, Phyllis Butow, Josephine Clayton, Joseph Coll, Jessica Cuddy, Fiona Davies, Haryana Dhillon, Mona Faris, Liesbeth Geerligs, Afaf Girgis, Peter Grimison, Tom Hack, Marnie Harris, Sharon He, Brian Kelly, Laura Kirsten, Toni Lindsay, Melanie Lovell, Tim Luckett, Lindy Masya, Michael Murphy, Jill Newby, Frances Orr, Alison Pearce, Don Piro, Nicole Rankin, Joanne Shaw, Tim Shaw, Heather Shepherd, John Stubbs, Rosalie Viney, Fiona White, Jackie Yim, Brian Kelly

**Affiliations:** 1 The University of Sydney, School of Psychology, Psycho-Oncology Co-operative Research Group (PoCoG), Chris O’Brien Lifehouse Level 6 (North), C39Z, Sydney, NSW 2006 Australia; 2 Chris O’Brien Lifehouse, Camperdown, NSW Australia; 3 The University of Sydney, Faculty of Medicine and Health, Sydney, NSW Australia; 4 The University of Sydney, Faculty of Medicine and Health, Research in Implementation Science and eHealth Group (RISE), Sydney, NSW 2006 Australia; 5 Northern Sydney Local Health District, St Leonards, NSW Australia; 6 University of Newcastle, School of Medicine and Public Health, Newcastle, NSW Australia

**Keywords:** Health professional education, Online modules, Communication skills, Acceptability, Real world implementation, Oncology

## Abstract

**Background:**

Anxiety and depression screening and management in cancer settings occurs inconsistently in Australia. We developed a clinical pathway (ADAPT CP) to promote standardized assessment and response to affected patients and enhance uptake of psychosocial interventions. Health professional education is a common strategy utilised to support implementation of practice change interventions. We developed an interactive on-line education program to support staff communication and confidence with anxiety/depression screening and referral prior to the ADAPT CP being implemented in 12 oncology services participating in the ADAPT CP cluster randomised controlled trial (CRCT). The aim of this research was to assess acceptability and uptake of the education program.

Patient Involvement: Although the wider ADAPT Program included patient consumers on the Steering Committee, in the context of this research consumer engagement included health professionals working in oncology. These consumers contributed to resource development.

**Methods:**

Development was informed by oncology and communication literature. The five online modules were pilot tested with 12 oncology nurses who participated in standardised medical simulations. Acceptability and uptake were assessed across the 12 Oncology services participating in the ADAPT CRCT.

**Results:**

During pilot testing the online training was reported to be acceptable and overall communication and confidence improved for all participants post training. However, during the ADAPT CRCT uptake was low (7%; *n* = 20). Although those who accessed the training reported it to be valuable, competing demands and the online format reportedly limited HPs’ capacity and willingness to undertake training.

**Conclusions:**

This interactive on-line training provides strategies and communication skills for front-line staff to guide important conversations about psychosocial screening and referral. Building workforce skills, knowledge and confidence is crucial for the successful implementation of practice change interventions. However, despite being acceptable during pilot testing, low uptake in real world settings highlights that organisational support and incentivisation for frontline staff to undertake training are critical for wider engagement. A multimodal approach to delivery of training to cater for staff preferences for face to face and/or online training may maximise uptake and increase effectiveness of training interventions.

**Trial registration:**

Pilot study ACTRN12616001490460 (27/10/2016). ADAPT RCT ACTRN12617000411347(22/03/2017).

**Supplementary Information:**

The online version contains supplementary material available at 10.1186/s12913-022-07521-5.

## Introduction

Clinically significant levels of psychological morbidity are reported in up to a third of all cancer patients [1]. Prevalence estimates for major depression (15%), minor depression or dysthymia (22%) and anxiety (10.3%) are also higher in cancer patients than those in the general population [2–4]. Inadequate treatment of anxiety and depression results in poorer cancer outcomes, lower quality of life and higher health service use [5–10]. There is a large evidence-base demonstrating psychological therapies, combined with medication where appropriate, are effective in improving outcomes of patients with anxiety and depression [11–13]. However, symptoms of anxiety and depression are often undetected and undertreated. This occurs because oncology health professionals may not recognise the symptoms, normalise patients’ distress or attribute psychological symptoms to cancer [14]. Patients are often not asked about their emotional distress and few patients volunteer this information during consultations, as they perceive it is not the role of medical staff to address emotional concerns, and some patients may decline referral or treatment for anxiety and depression [15–17].

To overcome these barriers and in recognition that psychosocial care is integral to cancer care, routine distress screening using validated screening measures is internationally endorsed [18]. The Psycho-oncology Co-operative Research Group (PoCoG) developed an evidence-based clinical pathway for the identification and management of anxiety and depression in adult cancer patients (ADAPT CP) [19]. The pathway, developed through synthesis of the current evidence and wide stakeholder consensus [20], provides recommendations to guide evidence-based practice using a stepped care model and outlines the roles and responsibilities of the clinical staff in identifying and managing anxiety and depression [19].

Common barriers reported by health professionals to initiating discussions about emotional concerns include perceived lack of time, insufficient training and lack of confidence [21, 22]. Therefore resources to support effective communication with patients to encourage anxiety/depression screening and referral, are critical to support implementation of the pathway into routine clinical practice. The aims of this research were to: (1) develop and then assess acceptability of an interactive on-line education program to support communication and increase confidence with anxiety/ depression screening and referral; and (2) assess the uptake of the training as a resource to support the implementation of the ADAPT CP across 12 Oncology services participating in the ADAPT Cluster randomised controlled trial (CRCT).

The CRCT evaluated the strategies required to achieve adherence to the recommendations of the ADAPT CP in routine clinical practice. The training modules formed part of a suite of resources that also included the ADAPT Portal (an online integrated pathway management system that tailored the ADAPT CP to local staff needs), patient education materials, and iCanADAPT, an online self-directed treatment program for patients. The resources were developed in response to a barrier analysis conducted to support implementation of the ADAPT CP [23].

## Methods

### Stage 1 development of the online training content using an iterative design methodology

A multidisciplinary expert advisory group (*n* = 7) with specialist clinical and research expertise in oncology, psycho-oncology, medical education, and communication was established to develop the content of the training program. Educational content was informed by a review of the oncology, nursing and communication literature and incorporated evidence-based recommendations as outlined in the ADAPT CP [19]. Training modules were developed using adult learning principles. To support face validity and modelling, video examples of clinical scenarios, peer to peer education and clinician perspectives were incorporated. To reinforce knowledge acquisition, self -reflective and evaluation exercises spaced throughout the training were included. Downloadable resources were included for future reference.

The communication components were developed based on the principles outlined in the Memorial Sloan–Kettering Comskil model [24]. Development of the training followed an iterative research methodology. The final training package comprised five modules. (Supplementary Table 1 summarises content of each module). The research team worked with web-designers at eviQ Education of the Cancer Institute NSW, a government initiative to provide on-line learning resources to oncology staff across Australia. The modules were hosted on the eviQ online training website.

Evaluation of the program was based on the Kirkpatrick model to evaluate the effectiveness of training [25]. The Kirkpatrick model is comprised of four levels that measure (1) the reaction of the participants (acceptability), (2) their learning (demonstration of skills and self -reported confidence), (3) their behaviour (uptake), and (4), impact on patient outcomes, although the last pillar was not evaluated as part of this research.

### Stage 2: pilot-testing of the on-line training – pre-post simulation study

#### Design

This was a pre-post training simulation study in which nurses completed questionnaires and a videoed encounter with an analogue patient before and immediately after completing training, followed by an interview to obtain their feedback.

#### Participants

Oncology nurses from two metropolitan hospitals in Sydney, Australia were invited to participate. Nurses self-selected after a presentation to the oncology nursing staff at each hospital. Written consent was obtained for all participants. This study received human research ethical approval (HREC/16/SVH/243).

#### Procedures

After providing written informed consent, participants completed the pre-training confidence questionnaire (described below). They were then asked to conduct an interaction with an analogue patient in a room set up as a clinic waiting room. Participants were informed that the duration of each simulated interaction was a maximum of 10-min, reflecting the time-limited nature of consultations. All participants participated in three scenarios (described below). After the pre-training simulation, participants were emailed a link to the on-line training and were given 3 weeks to complete the five modules. Participants then completed a post-training confidence and acceptability questionnaire and participated in three interactions. The interactions were video-recorded to facilitate their analysis. Immediately on completion of the post-training simulation session, participants were invited to participate in a face to face qualitative interview.

#### Medical scenarios and training of actors

Six medical scenarios were developed for the pre-post study. Cases involved cancer patients in active treatment attending a clinic appointment. The analogue patients displayed symptoms consistent with either anxiety or depression. The role of the nurse was to either (1) introduce routine screening, (2) make a referral for psychological assessment and ongoing management or (3) negotiate a referral with a patient who is reluctant to engage with psycho-oncology services. Six professional actors trained in medical simulation were given detailed dramaturgical instruction regarding their scenario. The order of presentation of the cases was randomly assigned for each nurse to reduce any order effects.

#### Measures

Demographic and nursing practice characteristics were collected to determine sample characteristics.

##### Acceptability

Post-training, participants were asked to rate their level of agreement on a 4-item 5-point Likert scale from *strongly disagree* (0) to *strongly agree* (5) with a series of statements related to whether the information provided, and skills taught were practical and useful, the course prompted reflection on current communication, and the module format and length were appropriate.

##### Communication

This was assessed from videoed scenarios using a standardised analysis framework developed by the researchers and based on the Comskil model and the communication goals of each of the three scenarios. The scoring assessment was based on the number of individual strategies and tasks outlined in the training. Each communication task was rated from 1 (not discussed) to 4 (fully discussed) and a total score for each interaction type was calculated with possible scores ranging from a minimum of 18 to maximum of 72 for screening, 22–88 for referral and 20–80 for negotiating a referral with a reluctant patient, with higher scores indicating better communication. Three trained coders blinded to timing of video-recording (i.e., pre or post training) independently coded the interactions (*n* = 72; 12 nurses). The coders underwent structured training and practiced coding until coding could be reliably applied. Inter-rated reliability based on Cohen’s kappa was also calculated.

##### Confidence

Pre-training confidence in identifying anxiety and depression, discussing anxiety and depression with patients, making a referral, and dealing with patients who are reluctant to take up referral, was assessed using a study-specific questionnaire (see Table 2). On the 5-item questionnaire, confidence was rated on a 5-point Likert scale from *not very confident* (0) *to very confident* (5), resulting in a maximum score of 20. Higher scores reflected greater confidence. Post-training, the same items were assessed, plus an additional item: confidence applying the anxiety and depression clinical pathway locally, scored as above.

##### Qualitative interviews

Immediately post training the nurses participated in a qualitative interview to explore their views about the training. The semi-structured interview schedule explored aspects of the training that participants found individually useful (or not), perceived usefulness of the training to clinical practice and general feedback on content and format.

#### Data analysis

Demographic and practice characteristics as well as training acceptability were summarised descriptively using SPSS (version 20; Chicago, Illinois). Within-group effect sizes (Hedges g) were calculated to measure effect size for the pre to post-training change. Interviews were recorded and transcribed verbatim. Thematic analysis was conducted using a content analysis approach [26]. The data were independently coded by two researchers to identify common themes.

### Stage 3: real world acceptability – uptake into clinical practice

The training modules were incorporated as a resource in the ADAPT CRCT and hosted on the Cancer Institute New South Wales EviQ website. Access was provided to health professionals at each site participating in the ADAPT CRCT. The ADAPT CRCT received Human Research Ethics approval (X16–0378 HREC/16/RPAH/522).

#### Participants

Twelve cancer services (7 urban and 5 regional) participated in the CRCT, which implemented and operationalised the ADAPT CP using an online portal (ADAPT Portal). Descriptive details of the cancer services are provided in Table 1. All health professionals within cancer services participating in the ADAPT CRCT were encouraged to undertake the online health professional training. The training was promoted during ADAPT Portal (*n* = 111 participants) and clinical pathway (*n* = 143 participants) training sessions prior to the CP implementation. Ongoing access to the training on the EviQ website was available to all staff registered as users in the ADAPT Portal (*n* = 302) through a link prominently displayed on the staff landing page.

**Table 1:** Site characteristics

Site ID	Site Location	Funding Type	No. of patients seen per 3-month period	No, of departments Included	Treatment modality departments Included	Tumour Streams Included	No. of streams included	FTE Psycho-social staff
1	Major city	Public	≥100	3	Med Oncology Rad Oncology Haematology	All	≥3	0.8
2	Inner regional	Public	< 100	3	Med Oncology Rad Oncology Haematology	All	≥3	0.6
3	Inner regional	Public	< 100	1	Med Oncology	All	≥3	0.6
4	Major city	Public	≥100	2	Med Oncology Surgical	Gastro -intestinal	1	2.4
5	Inner regional	Public	< 100	3	Med Oncology Rad Oncology Haematology	All	≥3	0
6	Major city	Public	≥100	2	Med oncology Haematology	All	≥3	7.9
7	Major city	Public	≥100	1	Surgical	Upper GI	1	2.4
8	Major city	Public	< 100	3	Med Oncology Rad Oncology Haematology	All	≥3	5
9	Major city	Public	≥100	1	Haematology	Lymphoma, acute leukemia, multiple myeloma	≥3	2.4
10	Major city	Public	≥100	3	Med Oncology Rad Oncology Surgical	Head & Neck	1	4
11	Major city	Public and Private	≥100	1	Med Oncology	Sarcoma, Gynae	2	6.9
12	Major city	Private	≥100	1	Med Oncology	All	≥3	0.9

#### Measures

##### Uptake

This was assessed as a ratio of the number of participants informed about the training, the number of participants who accessed the link to the training (based on page hits on the online training link within the ADAPT portal) as well as completion rate (defined as number of health professionals undertaking the training).

##### Qualitative interviews

Acceptability and appropriateness of the training were also explored within semi-structured interviews conducted at each site exploring staff perceptions of the resources more broadly.

#### Data analysis

Demographic and clinical characteristics as well as training uptake data were summarised descriptively. SPSS (version 20; Chicago, Illinois) was used to calculate all univariate statistics. Staff interviews were recorded, transcribed verbatim and coded in NVivo. Interview data were thematically analysed [27].

## Results

### Pilot-testing of the on-line training – pre-post simulation study

Of the 18 nurses who indicated initial interest, 13 consented to the study and participated in the pre-training simulations and 12 completed the training modules and completed the post-training simulations. Primary reasons for non-participation were a lack of time to schedule the simulations and concerns related to being evaluated in simulations. Participants were predominately female (*n* = 11) and experienced (> 5 years) in oncology (*n* = 9). Although no formal screening programs had been implemented at either hospital, five participants indicated screening for anxiety and depression was part of their current role, ten nurses reported their role encompassed referral of patients for psychosocial support and four nurses reported their role included provision of psychosocial support.

#### Assessing the effects of training

Table 2 presents the means and effect sizes for pre and post-training communication skills scores and consultation length. Inter-rater reliability for pre-training scores ranged from .566 (moderate) to .935 excellent and for post-training scores ranged from .859 (good) to .952 (excellent). Across the three consultations, communication improved from pre- to post training for all participants, although only *making a referral* demonstrated a large and significant within-group improvement between pre and post training (g = .83). Incorporation of the skills acquired through the training did not increase the length of consultations (Table 2), and there was a large and significant within-group decrease in consultation length when *making a referral* post-training (g = 1.17).

**Table 2:** Pilot-testing of the on-line training: Communication skills and consultation length changes pre-post training

	Pre-training	Post-training	Pre-post paired t-test		
	Mean (SD)	Mean (SD)	t	***p***-value	Hedges g
Communication Skills*					
Introducing screening	35.82 (8.18)	39.63 (7.80)	−1.33	.214	.46
Making a referral	36.41 (8.52)	43.54 (8.08)	−2.66	.024*	.83
Negotiating a referral	36.04 (9.18)	41.18 (8.82)	−1.77	.108	.55
Consultation Length **					
Introducing screening	7:57 (1.48)	8:42 (1:43)	−.024	.981	−.01
Making a referral	9.25 (.52)	7:35 (1:44)	3.26	.008***	1.17
Negotiating a referral	7:27 (1:35)	7:38 (2:04)	.603	.559	.25

*total score range18–72 introducing screening; 22–88 making a referral; 20–80 for negotiating a referral higher scores indicating better communication

** time in minutes

*** < .05;

#### Confidence ratings

Total self-assessed confidence increased from a pre-training mean score of 14.7 (SD 4.4) to 16.0 (SD 3.7) post-training. This increase was also observed for all tasks assessed, although only identifying anxiety symptoms was statistically significant (t = 2.2, *p* < .05). Scores for the additional post-training item indicated 63% (*n* = 7) participants were confident applying the ADAPT CP in their local institution. Mean item scores are listed in Table 3.

**Table 3:** Pilot-testing of the on-line training: participant confidence*

	Pre-training Mean (SD)	Post-training Mean (SD)
Identifying anxiety symptoms	2.92 (.90)	3.27 (1.00)**
Identifying depression symptoms	2.83 (.83)	3.09 (.83)
Discussing anxiety and depression screening with patients	2.75 (1.05)	2.91 (1.13)
Making a referral	3.83 (1.09)	3.91 (.94)
Negotiating with a patient who is reluctant to take up a referral	2.83 (1.03)	2.81 (.75)
**TOTAL confidence score**	**14.72 (4.38)**	**16.00 (3.74)**

*self-assessed rating from 0 – not at all confident to 5 very confident **p < .05

#### Acceptability

Qualitative feedback confirmed that the content of the online training was appropriate for oncology health professionals, and the format acceptable and engaging.

*I enjoyed getting the information from three different aspects, from the social worker, the psychiatrist, and psychologist … because they gave you a bit more information about how to approach thing …*. *I thought the scenario-based way it was set up was good (UPN3).*

*I loved the fact that it did guide you in the decision-making of the level of assistance [stepped care] that is required for each person. … I thought some of the skills with communication were excellent (UPN6).*

A few participants noted there was a lot of content, and finding time to complete the training was challenging.

*I think all of it was very good information, there was just a lot of it (UPN4).*

*it was probably … way too long. 4 h is a lot of time for people if you’re trying to do it during work. (UPN 2).*

More experienced participants perceived that the training would be of most benefit to those with less oncology experience.

*I think a lot of it I’ve done before myself, but I can imagine if you were in a different situation, if you’d just come into oncology, or you were on the wards, if you were not very experienced staff [the training would be helpful (UPN2).*

Overall participants viewed the training as positive and perceived they increased their knowledge and skills.

*It was quite an empowering 5 modules … I found the level of information, the videos, the short questions, actually quite useful. You think you’re doing it but when you do the questions, you can see how you can do better, you see how it can impact on a person. It made me identify the signs and symptoms of anxiety and depression a lot more than perhaps I would have prior. (UPN16).*

The qualitative data was supported by the post-training acceptability survey (Table 4), with 81.8% (*n* = 9) of participants indicating the information was practical and useful, enabled them to reflect on their practice (75%) and taught them additional skills (72.7%).

**Table 4:** Pilot-testing of the on-line training: Training Acceptability

	No participants Agree (n)	Percentage agreement
The training modules provided information which was practical and useful	9	81.8
The training modules taught me skills which were practical and useful	8	72.8
The training modules made me reflect on my current ways of communicating with patients about anxiety and depression screening and referral	9	75
The format of the training modules was appropriate	8	72.7
The length of time of training modules was appropriate	7	63.6

### Real world acceptability – uptake into clinical practice

Of the 286 active users registered within the ADAPT portal, 37 participants (12.9%) from across 11 of the 12 sites accessed the training via the link from the ADAPT portal. However only 20 of 37 (54%) of participants who accessed training completed the training modules, and only 4 of 37 (11%) completed the post-training assessment. Participants who accessed training included nurses (*n* = 13), social workers (*n* = 4) and clinical administration staff (*n* = 2), with two participant disciplines not reported. Forty-seven percent (*n* = 9) of those who accessed training indicated they had less than 2 years in oncology, although 42% (*n* = 8) of participants reported more than 10 years oncology experience. No data is available for those registered in the portal who did not access the training.

Analysis of the page clicks (*n* = 96) confirmed that participants accessed the training on multiple occasions throughout the ADAPT CRCT suggesting that the training was not completed in a single session but was accessed on an as needs basis.

#### Confidence ratings

Total self-assessed confidence increased from a pre-training mean score of 14.4 (SD 45.2) to 17.0 (SD 5.2) post-training. This increase was also observed for all tasks assessed, although the small number of post-training responses (*n* = 4) disallowed further analysis. Mean item scores are listed in Table 5.

**Table 5:** Real world Acceptability: Pre-post training self-assessed confidence*

	Pre-training Mean (SD)	Post-training Mean (SD)
Identifying anxiety symptoms	3.0 (1.23)	3.50 (1.29)
Identifying depression symptoms	3.0 (1.21)	3.50 (1.29)
Discussing anxiety and depression screening with patients	2.8 (1.10)	3.25 (0.96)
Making a referral	3.1 (1.25)	3.00 (0.82)
Negotiating with a patient who is reluctant to take up a referral	2.6 (1.14)	3.25 (1.71)
**TOTAL confidence score**	**14.74 (5.18)**	**17.00 (5.19)**

*self-assessed rating from 0 – not at all confident to 5 very confident **p < .05

#### Acceptability

Qualitative feedback exploring staff perceptions of the resources more broadly was obtained at three timepoints within the ADAPT CRCT (baseline (T0), 6 months (T1) and 12 months (T2) post implementation) and confirmed participants who accessed the training perceived it to be acceptable, appropriate, and informative. These interviews also confirmed that access continued for the 12 months implementation period rather than being limited to the study initiation phase.

*Oh, the eviQ stuff. Yeah, that was really good. I liked doing it actually, it just clarified a lot of things and gave me that reminder - - - about why we’re doing it, and also how to initiate it and bring it up in conversation and, − I mean, you’ve been nursing for so long and not incorporating it all that often. Like, it’s, it’s hard to change your practice --- so it was really helpful to actually get those skills and go through the scenarios as to how to incorporate screening and introducing it. (Nursing Staff, T0, S12PID01).*

*I found the resources ADAPT provided were really helpful to start off with, it … confirmed and enhanced your knowledge and experience in the areas. … I watched some of the … webinars... On EviQ. They were really helpful. (Nursing Staff, T1, S10PID02).*

*The online training was great. I did do quite a lot of it, and - - - I found it really wonderful. I highly recommend that side of the training.* (Psychosocial Staff, T2, S07PID05).

Other reported barriers to uptake included preference for face-to-face training, or a lack of protected time to undertake the online modules during work hours.

*I guess, different people have preference for different methods of learning but, … you could allocate 30 or 45 min … where people go to the education room and just have protected time to sit for that time and do it. … This idea that people would just do it in their own time online often means it doesn’t happen. So I think it’s certainly good and important to have it available, um, but, − the challenge is how to do it. (Medical Staff, T1, S11PID05).*

## Discussion

Effective communication in cancer care requires complex communication skills, which are essential for patient-centred care [28]. Inadequate communication can increase patient distress, [29] and conversely skilled and empathic communication and promotion of psycho-social care by oncology health professionals can help to overcome barriers to accessing psychological support, leading to improved patient outcomes [30, 31].

However discussing emotional concerns is reported to be challenging, in part because most clinicians have not received the formal evidence-based communication skills training they need to provide high-quality communication [32]. Nurses in particular report the need for training in communicating with patients about emotional concerns [33]. Given evidence that communication skills training is effective in increasing patient-centred care [34, 35], an interactive online training program for health professionals on how to discuss screening and management of anxiety and depression was developed and assessed.

The pilot pre-post simulation study demonstrated the program was effective in improving communication and increasing participant confidence. These results are in line with previously reported acceptability of online training to support routine screening and management of distress in the context of cancer in the Canadian context, [36] but no such training had been developed for the Australian context. In line with the roles and responsibilities outlined in the ADAPT CP, [19] the majority of course participants were nurses, as they are most likely to discuss screening and referral with patients. These nurse participants reported high acceptance of the training; other disciplines who participated also found the program acceptable. However, the high acceptability reported during the pilot study did not translate to wider uptake in the clinical setting, suggesting acceptability of the training more broadly was lower than expected. This was supported by our qualitative assessment of ADAPT resource uptake within the larger ADAPT CRCT, with a number of nurses interviewed reporting that they did not undertake the training as they perceived they already had the required experience and skills addressed in the training.

The online training was developed based on the key principles of the Comskil training program [24] and evaluation of the effectiveness of the training was guided by the Kirkpatrick model [25]. However, despite the use of these evidence-based principles in the development of the training and demonstrated effectiveness and acceptability in a pre-implementation pilot study, few health professionals accessed the training during the CRCT. Based on the results of this study, stakeholder advisory groups may not be sufficient to guide development, and future research aiming to implement online training programs need to consider wider engagement in the development phase to ensure the program meets the needs of end-users.

A key barrier highlighted by participants that influenced training uptake was organisational commitment to training. Such commitment needs to move beyond policy documents that espouse patient-centred care to concrete standards that prioritise core competencies of communication and protected time for staff to undertake the training. In this study, lack of protected time was relevant for nurses in particular, who reported workload and leadership reluctance to support training as limiting their willingness and capacity to complete the modules. This is consistent with the view more broadly that nurses receive fewer opportunities for professional development in relation to communication skills than do other disciplines in which the commitment to ongoing training is high [36]. Participants also reported the time commitment to complete the training discouraged them from undertaking the modules in personal time as they needed to prioritise other mandatory training requirements over communication skills training. The lack of organisational support for training impacts not only on staff morale and levels of burnout [37] but negatively impacts clinical efficiency and patient outcomes [37]. Organisational commitment to improving communication is a key driver of communication skills training [38].

Traditional methods of communication skills training involving immersive workshops, frequently as part of residential or multi-day programs, although effective, are costly in terms of financial and staff resources, making wider inter-disciplinary implementation challenging [34]. The move to online learning in the health sector was perceived as a means of enabling staff to access learning opportunities at times and places that best fit in with their work and lifestyle [39]. Completion of online training is reportedly six times higher than face to face training, [40] with a recent systematic review finding higher self-assessed communication skills, objective knowledge and confidence after online training [41]. The module format of our training was designed to facilitate progress through the training in a staged way, with the ability to revisit components of the training as needed. Participants reported using the training across the 12-month implementation of the ADAPT CP and valued the ability to dip in and out of the training. However, the results of our study also highlight that consistent with previous research [41, 42], for some, the online delivery format was less appealing due to the lack of synchronous interaction and individualised feedback. Future strategies to improve communication skills training could include offering a blend of workshops and online learning tailored to staff preferences, providing protected time in a designated education room within the workplace to complete online learning, and providing incentives to complete training such as accreditation for continuous professional development. The development of our online education modules was based on a barrier analysis highlighting the need for greater training. However, the analysis did not identify online as the preferred mode of delivery. In hindsight, exploring end user preferences for training may have identified a preference for more interactive training options. Prior to undertaking the development of future online education programs, researchers may consider conducting a more detailed needs assessment to identify not only delivery preferences but more targeted training to meet self-identified gaps in knowledge, thereby streamlining the training.

Based on the qualitative interviews, clinicians who undertook the training as part of the ADAPT CRCT found the training useful and did not highlight concerns about the training length or content, although those who failed to engage with the training may have perceived the training required significant time commitments, given they were not provided information about the training length prior to opening the EviQ link. However, interview participants reported the lack of engagement was primarily due to lack of time to complete non-mandatory training without protected time, which is an organisational issue of workload, and/or a preference for face to face training, particularly for communication skills. Decisions to undertake training were reportedly based on capacity; mandatory training, which is linked to ongoing employment, is prioritised over personal professional development. This suggests that researchers consider the value of online training to both end users and organisations and weigh up whether the investment in time by staff to complete complex communication training is supported by the health system. Greater engagement at an organisational level to guide development may have increased participants’ commitment to facilitating professional development of their staff. However even with protected time and organisational commitment, if staff are not personally committed to improving communication, they may complete training just to ‘tick the box’, without truly engaging in the content. Therefore, strategies to engage staff at the individual level, as well as management at the service level, are required.

The results of this research need to be considered in light of a number of limitations. While all training modules were used by nurses and reported to be useful, no information was collected about the length, frequency, and duration of their use. The limited analytics of wider uptake also does not provide any indication related to whether the training was effective in changing objective communication skill behaviours and therefore, further research to assess the impact of communication skills training on patient outcomes including referral to and uptake of psycho-oncology interventions is required. Participants in the initial pilot study may not have reflected the wider oncology health professional population in their interest in undertaking online communication skills training, meaning our initial results were biased by self-selection. Finally, the collection of training uptake and acceptability data was limited to the health professional data collected as part of the main CRCT. This prevented us exploring potential differences between those who did and did not access the training and clinical experience.

## Conclusions

In summary, although online communication skills training to support the implementation of the ADAPT CP was effective in improving communication and increasing confidence in a research context, acceptability was not reflected in uptake more widely. Further implementation research to explore barriers and facilitators to uptake such as the impact of greater institutional support and incentivisation as well as multi-modal training programs may be required.

## Supplementary Information

**Additional file 1:** **Table 1.** Overview of Module Content.

## Data Availability

All data and materials can be requested by contacting the Corresponding author.

## Abbreviations

ADAPT CP: The Australian Clinical Pathway for the Screening, Assessment and Management of Anxiety and Depression in Adult Cancer Patients
ADAPT CRCT: ADAPT cluster randomised controlled trial
Comskil: Memorial Sloan Kettering Communication Skills Training Program
eviQ: Free resource of evidence-based, consensus driven cancer treatment protocols and information for use at the point of care hosted by Cancer Institute NSW
iCanADAPT: Online transdiagnostic online cognitive behavioural therapy
PoCoG: Psycho-oncology Co-operative Research Group
SD: Standard deviation
