# Supplementary material for: Development, acceptability and uptake of an on-line communication skills education program targeting challenging conversations for oncology health professionals related to identifying and responding to anxiety and depression
Source: BMC Health Serv Res. 2022 Jan 31;22:132. doi: 10.1186/s12913-022-07521-5 (PMC8805223; doi:10.1186/s12913-022-07521-5)
Supplement: Supplementary file 1 — Additional file 1: Table 1. Overview of Module Content. [file 12913_2022_7521_MOESM1_ESM.docx]

Table 1: Overview of Module Content

| **Modules** | **Core Content** |
| --- | --- |
| 1. Anxiety and Depression in Cancer | - Understanding the difference between general   distress and anxiety and depression |
|  | - Identifying anxiety and depression symptoms and risk factors |
| 1. A Stepped Care Model | - Routine screening for anxiety and depression in   cancer |
|  | - Screening measures for anxiety and depression |
|  | - Assessment for anxiety and depression |
|  | - The stepped care model of care for  management of patients with anxiety and depression |
|  | - Professional roles and responsibilities for the management of anxiety and depression in cancer |
| 1. Initiating a conversation about   routine screening | - Framing the screening conversation |
|  | - Empathising with patient experience |
|  | - Identifying sources of potential anxiety and depression |
|  | - Educating patients about anxiety and depression and support available |
|  | - Discussing next steps with patients |
| 1. Initiating a conversation about   referral | - Initiate a conversation  recommending referral for formal psychological assessment  and additional   psychosocial support |
|  | - Convey empathy about the patient’s experience |
|  | - Clarify patient symptoms and safety risk; use the stepped care model to triage patients for formal psychological assessment |
|  | - Discuss options for provision of psychological care and patient preferences for support |
|  | - Confidently recommend a referral to specialist  psychological services |
|  | - Discuss next steps for referral uptake |
| 1. Managing Declining Referral in at Risk Patients and other Challenging Conversations | - Common reasons why patients decline offers of psychological support |
|  | - Assessing safety risk |
|  | - Revisiting recommendations for support |
|  | - Management of patients who decline offers of psychological support |
|  | - Dealing with patients at risk of self-harm |
